# Temporal Relationship between Atrial Fibrillation and Heart Failure Development Analysis from a Nationwide Database

**DOI:** 10.3390/jcm10215101

**Published:** 2021-10-30

**Authors:** Yves Cottin, Brahim Maalem Ben Messaoud, Antoine Monin, Pierre Guilleminot, Arnaud Bisson, Jean-Christophe Eicher, Alexandre Bodin, Julien Herbert, Yves Juillière, Marianne Zeller, Laurent Fauchier

**Affiliations:** 1 Cardiology Department, University Hospital Center Dijon-Bourgogne, 21000 Dijon, France; brahimsap@hotmail.fr (B.M.B.M.); antoine.monin@chu-dijon.fr (A.M.); pierre.guilleminot@chu-dijon.fr (P.G.); jean-christophe.eicher@chu-dijon.fr (J.-C.E.); 2 Cardiology Department, University Hospital Center Trousseau and University François Rabelais, 37000 Tours, France; arnaud.bisson37@gmail.com (A.B.); a.bodin@chu-tours.fr (A.B.); j.herbert@chu-tours.fr (J.H.); laurent.fauchier@univ-tours.fr (L.F.); 3 Cardiology Department, University Hospital Center, 54000 Nancy, France; yves.juilliere@wanadoo.fr; 4 Physiopathologie et Epidémiologie Cérébro-Cardiovasculaires (PEC2), Equipe d’Accueil (EA 7460), University of Bourgogne Franche Comté, 21000 Dijon, France; marianne.zeller@u-bourgogne.fr

**Keywords:** atrial fibrillation, heart failure, prognosis

## Abstract

Background Atrial fibrillation (AF) and heart failure (HF) often co-exist and are closely intertwined, each condition worsening the other. The temporal relationships between these two disorders have not yet been fully explored. We aimed to address the outcomes of patients hospitalized with HF and AF based on the chronology of the onset of the two disorders. Methods From the administrative database for the whole French population, we identified 1,349,638 patients diagnosed with both AF and HF between 2010 and 2018; 956,086 of these AF patients developed HF first (prevalent HF), and 393,552 developed HF after AF (incident HF). The outcome analysis (all-cause death, cardiovascular (CV) death, ischemic stroke or hospitalization for HF) was performed with follow-up starting at the time of last event between AF or HF in the whole cohort and in 427,848 propensity score-matched patients. Results During follow-up (mean follow-up 1.6 ± 1.9 year), matched patients with prevalent HF had a higher risk of all-cause death (21.6 vs. 19.3%/year, hazard ratio (HR) 1.10, 95% CI 1.08–1.11), CV death (7.7 vs. 6.5%/year, HR 1.14, 95% CI 1.12–1.16) as well as re-hospitalization for HF (19.4 vs. 13.2%/year, HR 1.44, 95% CI 1.41–1.46) than those with incident HF. The risk for ischemic stroke was lower in prevalent HF than in incident HF (1.2 vs. 2.4%/year, HR 0.50, 95% CI 0.48–0.52). Conclusions We identified two distinct clinical entities: patients in whom HF preceded AF (prevalent HF) had higher mortality and higher risk of re-hospitalization for HF.

## 1. Introduction

Atrial fibrillation (AF) and heart failure (HF) are two cardiovascular epidemics [1,2,3]. In patients with AF, the most common cause of death is cardiovascular and, in particular, through HF [4,5]. AF and HF often co-exist in a reciprocal relationship where the occurrence of AF increases with the severity of HF [6]. Taken separately, these two diseases worsen patient prognosis, and the prognosis is further degraded when both conditions are present [7,8,9]. According to the literature, HF is recorded in 34% of AF patients, and AF is recorded in 42% of HF patients [10]. Therefore, there is a reciprocal interplay between these diseases, with one aggravating the other and vice versa in a vicious circle [10,11]. Small cohort studies have suggested a worse prognosis in patients who have prevalent HF [12]. However, the temporal relationship between the two conditions has not yet been fully explored. The aim of the present study was to assess, on a national scale, the prognosis of patients hospitalized with HF and AF based on the chronology of the onset of the two disorders.

## 2. Methods

### 2.1. Study Design 

This retrospective cohort study was based on data from the French administrative hospital discharge database (Programme de Médicalisation des Systèmes d’Information; PMSI), which collects collected medical information according to the International Classification of Diseases, Tenth Revision (ICD-10), and medical procedures recorded according to the national nomenclature Classification Commune des Actes Medicaux (CCAM). The reliability of PMSI data has already been assessed, and this database has previously been used to study patients with cardiovascular conditions, including AF and HF [13,14,15]. Use of medication was identified from a 1/97 permanent random sample of the complete French nationwide claims database (Echantillon Généraliste de Bénéficiaires, EGB—general sample of healthcare beneficiary. This sampling procedure has been previously used in patients with cardiac conditions with similar inclusion criteria (patients with AF and HF) [16,17,18,19]. Patients were considered to be included in a treatment group for a given class of drugs if they received a treatment from that class for ≥60 days within 6 months after discharge for the last event between AF or HF. The French Data Protection Authority granted access to the PMSI data, and procedures for data collection and management were approved by the independent national ethics committee for human rights in France (Commission Nationale de l’Informatique et des Libertés; CNIL), which ensures that all information is kept confidential and anonymous in compliance with the Declaration of Helsinki (authorization number 1897139).

### 2.2. Study Population

**Figure 1:**
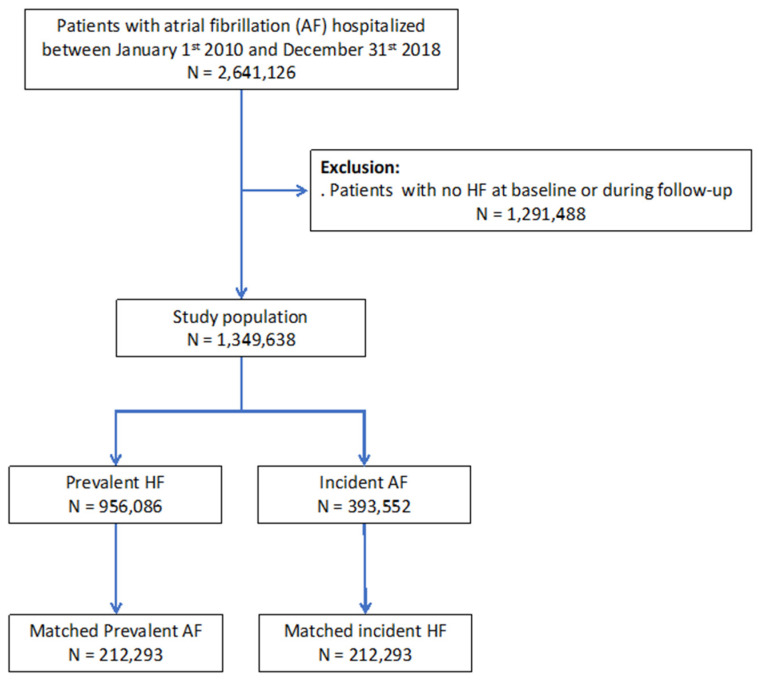
Flowchart of the study population and analyses.

From January 2010 to December 2018, 2,641,126 adults (age ≥ 18 years) were hospitalized with a diagnosis of AF as the principal diagnosis (i.e., the condition justifying hospital admission), a related diagnosis, or a significantly associated diagnosis (i.e., co-morbidity or associated complication). Starting from this database of patients with AF, we extracted patients with HF using the same principle, allowing us to identify our cohort of interest of 1,349,638 patients with a diagnosis of both AF and HF (Figure 1). We defined patients with prevalent HF as those who had HF diagnosed earlier than AF. We defined patients with incident HF as those who had AF diagnosed earlier than HF. Those with AF and HF on the same day were not considered as incident HF during follow-up. These patients with AF and HF simultaneously at baseline were considered as having history of HF (prevalent HF), since it was likely that most of these individuals were previous outpatients with structural heart disease or patients with recent HF as part of tachycardiomyopathy who cannot be considered as patients with AF alone [20]. For each hospital stay, we evaluated a proxy of the HAS-BLED score (i.e., all items except labile INR, which was unavailable, as in most administrative databases using ICD codes) and evaluated the CHA_2_DS_2_-VASc score. We performed an analysis with matching of patients when incident HF was registered, a referent characterized by prevalent HF at the diagnosis of AF being matched for the propensity score, with follow-up starting at the time of last event between AF or HF.

### 2.3. Outcomes

We aimed to evaluate the incidence of all-cause death, cardiovascular death, non-cardiovascular death, ischemic stroke and re-hospitalization for HF (identified as the main diagnosis for hospital stay). To enhance the validity of our analysis, we also evaluated incidence rates of non-cardiovascular death and cancer as control endpoints. The endpoints were evaluated from January 2010 according to follow-up starting at the time of last event between AF or HF until each specific outcome or the last recorded follow-up in the absence of an outcome. Information on outcomes during follow-up was obtained by analyzing the PMSI codes for each patient. The cause of death was identified based on the main diagnosis during the hospital stay that resulted in death.

### 2.4. Statistical Analysis 

**Figure 2:**
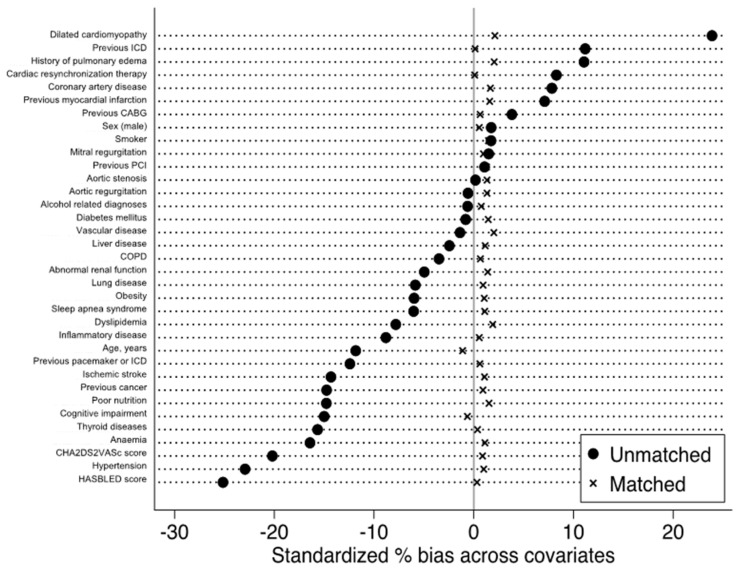
Standardized percentages of bias across main baseline characteristics in unmatched and matched patients with AF and HF, with incident HF and prevalent HF.

**Figure 3:**
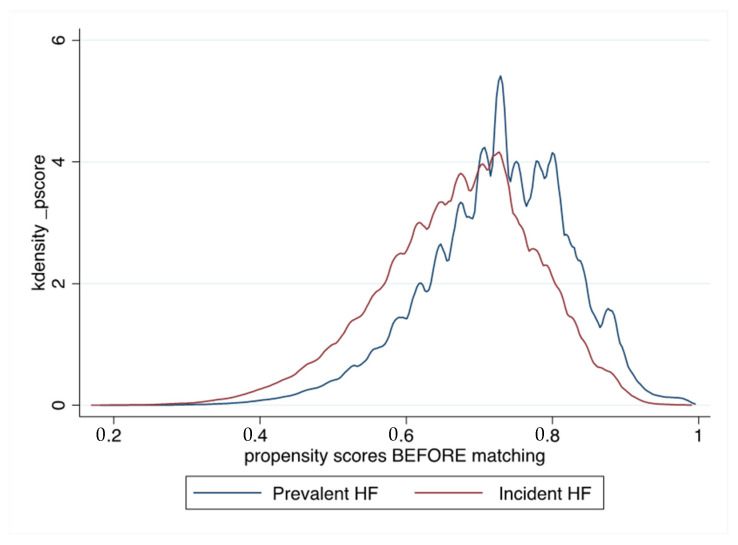

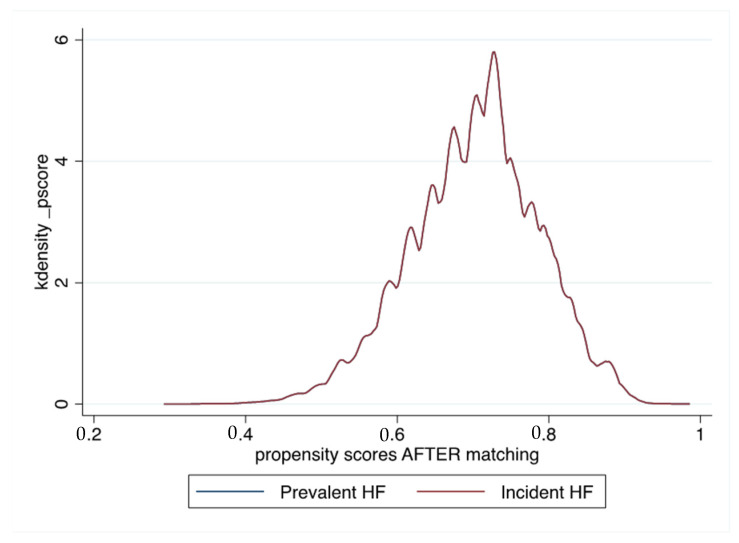
Propensity score distribution for unmatched and matched populations of patients with AF and HF, with incident HF and prevalent HF.

Qualitative variables are described as frequencies and percentages, and quantitative variables as means (standard deviations (SD)). Owing to the non-randomized nature of the study, and considering significant differences in baseline characteristics, propensity score matching was used to control for potential confounders of the treatment–outcome relationship. Propensity scores were calculated using logistic regression with history of HF at the time of AF (prevalent HF, i.e., HF previous or simultaneous to AF) as the dependent variable. The propensity score calculation included baseline characteristics listed in Table 1. Each AF patient with prevalent HF was matched with a patient with incident HF (1:1) using the one-to-one nearest neighbor method (with a caliper of 0.001 of the SD of the propensity score on the logit scale) and no replacement (Figure 2 and Figure 3). To test for matching procedure quality, we assessed the distribution of demographic data and co-morbidities in the AF first (incident HF) and HF first (prevalent HF) unmatched and matched cohorts with standardized differences, which were calculated as the difference in the means or proportions of a variable divided by a pooled estimate of the SD of that variable (proportion being considered as a continuous variable ranging from 0 to 1). For the outcomes analysis in the matched cohort, the incidence rates (%/year) for each outcome of interest during follow-up was estimated in the incident HF and prevalent HF groups and compared using hazard ratios (HR). We calculated delta CHA_2_DS_2_-VASc score in each patient for evaluating incident co-morbidities during follow-up [19]. *p* values are reported without and with correction for multiple comparisons using Bonferroni correction and considering 4 different comparisons. All comparisons with *p* < 0.05 were considered statistically significant. A two-sided *p* value of <0.05 was considered statistically significant. All analyses were performed using Enterprise Guide 7.1, (SAS Institute Inc., SAS Campus Drive, Cary, NC, USA) and STATA version 16.0 (Stata Corp, College Station, TX, USA).

## 3. Results

### 3.1. Baseline Characteristics

Overall, we identified 1,349,638 patients in the database between January 2010 and December 2018, including 393,552 patients (29.2%) with incident HF and 956,086 patients (70.8%) with prevalent HF (Table 1). In the unmatched population, patients with prevalent HF patients were slightly younger, less likely to have cardiovascular and non-cardiovascular disease and lower CHA_2_DS_2_-VASc and HASBLED scores. The average time between hospitalization with HF and evidence of AF in the prevalent HF patients (excluding those with AF and HF diagnosed during the same hospitalization) was 772 ± 778 days (median 516, IQR 126–1194 days). The average duration between hospitalization with AF and re-hospitalization with HF in the incident HF patients was 720 ± 717 days (median 481, IQR 120–1131 days, *p* < 00001). The percentages of use of each medication of interest are presented in Table 2. They were similar in both groups, although patients with prevalent HF had a slightly lower use of ACEi/ARBs than those with incident HF. There was sub-optimal use of oral anti-coagulation (around 50–55% with VKA or NOAC in both groups, around 85% for anti-thrombotic therapy including anti-platelet therapy). Use of ACEi/ARB and beta-blockers was rather low (40–50%). Table 3 shows baseline characteristics after matching. Standardized differences in matched patients were <5% for all parameters and generally < 2%.

### 3.2. Clinical Outcomes in the Unmatched Cohort 

During follow-up in the unmatched population (mean 1.7 ± 2.0 years; median 0.8, IQR 0.1–2.7 years), the CHA_2_DS_2_-VASc score increased, with a mean Delta CHA_2_DS_2_-VASc score of 0.26 in patients with prevalent HF and 0.35 in those with incident HF. Risk of all-cause death was lower in the prevalent HF group, with an incidence rate of 19.0 vs. 21.8%/year (Table 4). The incidence of cardiovascular death was slightly higher in the prevalent HF group, although non-cardiovascular death was lower. The rate of re-hospitalization for HF was higher in the prevalent HF group. By contrast, the risk of ischemic stroke was slightly lower in the prevalent HF (Table 3).

### 3.3. Clinical Outcomes in the Matched Cohort 

**Figure 4:**
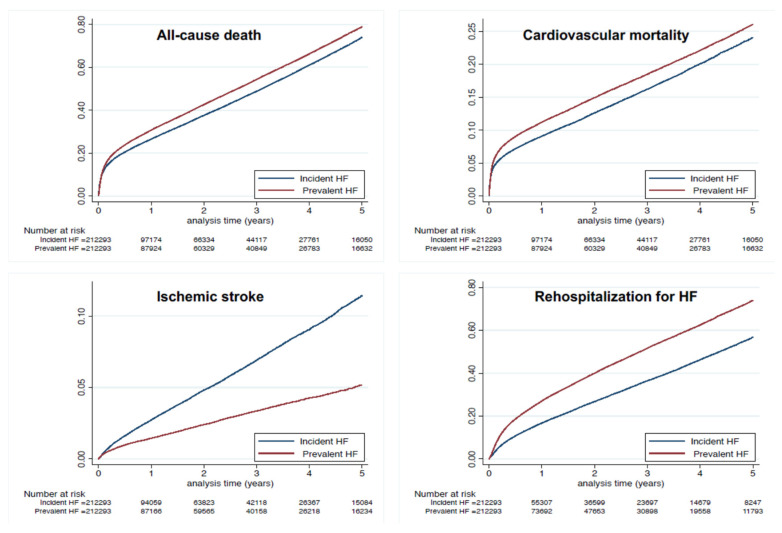
Cumulative incidences for all-cause death (**top left panel**), cardiovascular death (**top right panel**) and ischemic stroke (**lower left panel**) and re-hospitalization for HF (**lower right panel**) in the matched cohort of patients with AF and HF, with incident HF and prevalent HF.

During follow-up in the propensity score-matched population (mean 1.6 ± 1.9 years; median 0.7, IQR 0.1–2.5 years), the CHA_2_DS_2_-VASc score increased with a mean Delta CHA_2_DS_2_-VASc score of 0.12 and 0.28 in matched patients with prevalent HF and incident HF, respectively (*p* < 0.0001). Risk of all-cause death was higher in the prevalent HF group, with an incidence rate of 21.6 vs. 19.3%/year (Table 5 and Figure 1). The incidence of cardiovascular death was also higher in the prevalent HF group (incidence rate 7.7 vs. 6.5%/year), as was non-cardiovascular death (Table 5 and Figure 4). The risk for ischemic stroke was markedly lower in the prevalent HF group (incidence rate 1.2 vs. 2.4%/year). The prevalent HF group in the matched cohort by contrast had a higher risk of re-hospitalization for HF, with an incidence rate of 19.4 vs. 13.2% (Table 5 and Figure 4).

### 3.4. Outcomes in Patients with (1) Prevalent HF, (2) AF and HF Diagnosed during the Same Hospitalization or (3) Incident HF

**Figure 5:**
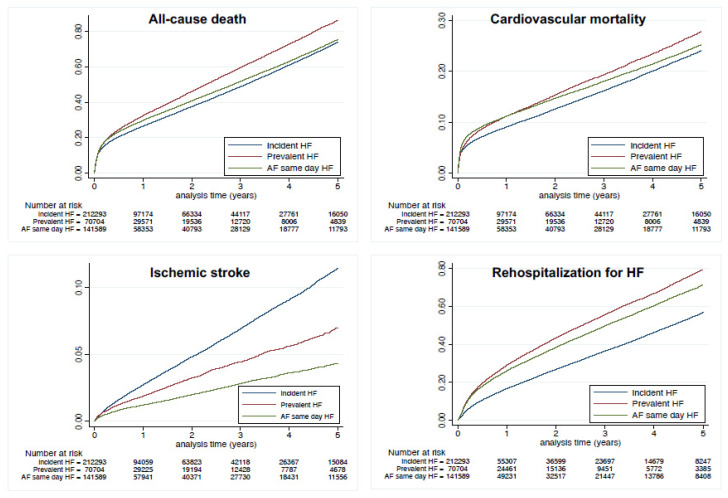
Cumulative incidences for all-cause death (**top left panel**), cardiovascular death (**top right panel**), ischemic stroke (**lower left panel**) and re-hospitalization for HF (**lower right panel**) in the matched cohorts of patients with (1) incident HF, (2) prevalent HF or (3) AF and HF diagnosed during the same hospitalization (follow-up starting at the time of AF). Compared to prevalent HF (i.e., HF earlier than AF) as the reference, hazard ratio was 0.90 (0.88–0.91) for AF and HF diagnosed during the same hospitalization and 0.85 (0.84–0.86) for incident HF when analyzing the risk of all-cause death; 0.98 (0.95–1.00) and 0.86 (0.84–0.88), respectively, when analyzing the risk of cardiovascular death; 0.64 (0.60–0.69) and 2.44 (2.32–2.57), respectively, when analyzing the risk of ischemic stroke; and 0.92 (0.90–0.93) and 2.26 (2.22–2.29) respectively when analyzing the risk of re-hospitalization for HF.

Considering our hypothesis that patients with AF and HF diagnosed on the same day were considered as having prevalent HF, we performed a sensitivity analysis in matched patients divided into three groups of patients: (1) those with prevalent HF earlier than AF, (2) those with AF and HF diagnosed on the same day and (3) those with incident HF. The outcomes are displayed on Figure 5. Incidences for outcomes in patients with AF and HF diagnosed on the same day were (with different aspects) closer to those seen in patients with HF first than to patients with incident HF.

## 4. Discussion

In this large nationwide study, two distinct clinical entities were distinguished based on the chronological sequence of AF and HF onset. Our results indicated that: (1) most patients hospitalized with AF had HF before AF (prevalent HF); (2) in the propensity score-matched population, AF patients who had prevalent HF had a higher incidence of all-cause death including cardiovascular death than those with incident HF; (3) in the propensity score-matched population, patients with prevalent HF had a higher incidence of re-hospitalization for HF and a lower risk of ischemic stroke than those with incident HF.

### 4.1. Study Population with AF and HF

Our large database study included patients hospitalized with both AF and HF diagnosis. In 346 ambulatory patients with newly developed AF that were followed up for 12 years [21], approximately 10 years after inclusion, only 4% of patients had presented with HF. In a small study including only hospitalized patients with AF and HF (*n* = 182), those who developed AF before or as the same time as HF (“incident HF”) (137 patients) were compared with patients who had developed HF before AF (“prevalent HF”) (45 patients) [6].

The Framingham Heart study included 1737 outpatients with new-onset AF or HF between 1980 and 2012 [22]. Among patients with new AF, more than one-third (37%) had HF and, conversely, among 1166 individuals with new HF, more than half (57%) had AF [20]. In the larger study by McManus et al., including 23,644 patients with HF, 11,429 (48.3%) had documented AF [23], and one-third of patients with HF with reduced ejection fraction (HFrEF) had pre-existing AF. The frequency of pre-existing AF among patients with HF with preserved ejection fraction (HFpEF) was 43.2%. Our study is, thus, by far the largest analysis of unselected patients with AF and HF and provides contemporary insight on these epidemiological aspects.

### 4.2. Temporality between Onset of Atrial Fibrillation and Heart Failure 

In our population, patients were readmitted for HF with a yearly rate of 15–20%. This rate is high compared with previous works [21] on patients with “lone AF”, in which only 4.0% of patients developed HF at 2 years. However, their study population had less severe disease and was considerably younger: 43 years on average compared to 79 years in our cohort, which is more likely to represent the full picture of patients with AF and HF [21]. In a recent analysis, 21% of patients classified as incident HF developed HF over 18 months of follow-up [6]. In the Framingham Heart study patients, AF occurred in more than half of individuals with HF, and HF occurred in more than one third of individuals with AF. AF may precede and follow HF with both preserved and reduced ejection fraction, with some differences in temporal association and prognosis [22]. Our study provides important information regarding the time to hospital re-admission. The average period of time between hospital admission for AF and re-hospitalization for HF was 720 days, and the average time between hospital admission for HF and re-hospitalization with evidence of AF was 772 days.

The findings that incidences for outcomes in patients with AF and HF diagnosed on the same day were closer to those seen in patients with HF first than to patients with incident HF may confirm our hypothesis in the methods that a significant part of these patients may have been previous outpatients with structural heart disease or patients with recent HF as part of tachycardiomyopathy.

### 4.3. All-Cause Death and Cardiovascular Death

Patients with AF and previous (prevalent) HF were slightly younger and had a lower risk of all-cause death than those with incident HF in the unmatched analysis but a higher risk of death in the propensity matched analysis. Our results for all-cause and cardiovascular mortality highlight the negative effect of prevalent HF on the prognosis of patients with AF and the need for an early and optimal management of AF in these patients. We had no information regarding ejection fraction in our analysis. Though the methodology was very different, Santhanakrishan et al. found that both AF and HF indicated greater mortality risk compared with just one condition, particularly among individuals with new HFrEF vs. new HFpEF associated with prevalent AF [22]. When HFpEF is as common as HFrEF, both prevalent and incident AF were associated with increased mortality in HFpEF (HR 1.30 and 2.45, respectively, compared with no AF) [24]. Use of ACEi/ARB and beta-blockers in our population seemed sub-optimal in the analysis of the EGB sample, in agreement with previous works [18]. It should, however, be noticed that these treatments may not be systematically indicated in HF patients with preserved EF, which may represent a significant proportion of AF patients in our analysis.

In a systematic review and meta-analysis of 10 studies in patients with both AF and HFpEF, all-cause mortality was significantly higher in AF–HFrEF (RR 1.24, 95% CI 1.12–1.36; *n* = 45,100), with absolute death rates of 24% compared with 18% in AF–HFpEF over 2 years. There were no significant differences in incident stroke or hospital admission for HF [25]. In the large cohort from the European registry, the authors found that the risk associated with AF for all-cause death was 0.92 (95% CI 0.782–1.091) in HFrEF (<40%) and was 1.20 (95% CI 0.954–1.504) in HFpEF (≥50%) [26].

### 4.4. Higher Incidence of Ischemic Stroke in Patients Hospitalized with “Incident HF” 

Although patients with prevalent HF had higher prevalence of CAD and dilated cardiomyopathy, they had slightly lower CHA_2_DS_2_-VASc score in the unmatched analysis. In the matched analysis (resulting in a similar CHA_2_DS_2_-VASc score in both groups), we interestingly found a higher risk of ischemic stroke in the incident HF group. CHA_2_DS_2_-VASc scores and stroke risk was non-static, and several patients had ≥1 new stroke risk factor(s) during follow-up. This was more frequent in patients with incident HF when one considered Delta CHA_2_DS_2_-VASc score in the matched cohort. This may, in part, explain why stroke risk was different in patients with incident HF and prevalent HF, and this also highlights that the follow-up CHA_2_DS_2_-VASc score, and its change may also play a role when studying risk of ischemic stroke in addition to the baseline CHA_2_DS_2_-VASc score. Risk of stroke was comparable in patients with HF and without HF [10] and whether the ejection fraction was altered or not. No stroke was recorded in the incident HF or prevalent HF group after 18 months of follow-up [6]. Our large analysis at a nationwide level thus adds new insights regarding association of AF and HF with the risk of ischemic stroke.

Several hypotheses may explain the higher rate of ischemic stroke in the incident HF group: (1) a poorer follow-up than for patients with prevalent HF, (2) a lower proportion of anti-coagulant treatment (which was not included in our matched analysis), (3) variations in appendage morphology and volume in case of HF with changes in atrial pressure, (4) a poor adherence to pharmacological therapies, (5) different types of AF (permanent or paroxysmal) and (6) a higher risk of unknown incident co-morbidities, as mentioned above.

### 4.5. Limitations

The main limitation is inherent to the retrospective, observational nature of the study and its potential biases. The diagnoses and occurrence of outcomes in our study were based on the diagnostic codes registered by a responsible physician and were not further checked externally. However, as coding of complications is linked to reimbursement and is regularly controlled, it is expected to be of good quality. We had no information for death occurring outside hospitals. Our large population of patients hospitalized with AF and HF likely represents a heterogeneous group of patients admitted with various kinds of illnesses and severities, which may have affected prognosis. Some data were unavailable for evaluating the HF severity such as LVEF, NYHA class or BNP level. AF pattern (paroxysmal, persistent or permanent) have been included in ICD codes in recent years, but they are not commonly used and are possibly unreliable since identification of these AF patterns are not so well-known in the medical community. Another limitation is the lack of information on anti-thrombotic drug use and its possible changes during follow-up, as data regarding these therapies were not available in the complete database. There is a similar issue regarding the lack of information in terms of therapies recommended for HF and AF beyond the representative sample from our analysis. We did not analyze doses, which is beyond the scope of this study. The impact of VKA use also depends on TTR, which was not available in our dataset. Further, the non-randomized design of the analysis leaves a risk of residual confounding factors. Definite conclusions for comparisons between groups may not be fully appropriate even though multi-variable matching was done, as it cannot fully eradicate the possible confounding variables between these groups. The study was not racially and ethnically diverse, and our findings may not be generalizable to other populations.

## 5. Conclusions

We identified two distinct clinical entities based on the chronological sequence of AF and HF onset in a national cohort of patients hospitalized with both AF and HF. Our data highlight dissimilarities in the risk of clinical events for these associated diseases according to their time of onset. It is, therefore, better to separate patients with AF and HF into two distinct groups that may not be part of the same entity. Our results indicate that HF preceding AF (prevalent HF) was worse for the risk of death than the opposite, which might have implications for the treatment and follow-up of affected patients. Further studies investigating the underlying mechanisms and the interplay between these two conditions are warranted. Optimal management of AF should take into account all underlying heart diseases and related co-morbidities, not just focusing on preventing strokes at the expense of preventing or treating HF. In order to reduce mortality, therapeutics should be carefully targeted for treating as early as possible the whole clinical picture in patients with AF.

## Figures and Tables

**Table 1:** Baseline characteristics of unmatched patients with AF with prevalent HF or incident HF (follow-up starting at the time of last event between AF or HF).

	Prevalent HF	Incident HF	Standardized Difference, Prevalent HF vs. Incident HF	Total
	(*n* = 956,086)	(*n* = 393,552)	(%)	(*n* = 1,349,638)
Age, years	79.4 ± 11.2	80.7 ± 10.2	−11.8	79.8 ± 10.9
Sex (male)	506,100 (52.9)	204,887 (52.1)	−20.2	710,987 (52.7)
CHA_2_DS_2_-VASc score	4.4 ± 1.4	4.7 ± 1.3	−25.1	4.5 ± 1.4
HASBLED score	1.9 ± 0.8	2.1 ± 0.8	1.7	1.9 ± 0.8
Hypertension	651,087 (68.1)	307,749 (78.2)	−22.9	958,836 (71.0)
Diabetes mellitus	255,132 (26.7)	106,430 (27.0)	−0.8	361,562 (26.8)
Smoker	75,809 (7.9)	29,387 (7.5)	1.7	105,196 (7.8)
Dyslipidemia	255,314 (26.7)	118,970 (30.2)	−7.8	374,284 (27.7)
Obesity	173,860 (18.2)	80,859 (20.5)	−6.0	254,719 (18.9)
History of pulmonary edema	65,725 (6.9)	17,064 (4.3)	11.1	82,789 (6.1)
Dilated cardiomyopathy	184,186 (19.3)	42,456 (10.8)	23.9	226,642 (16.8)
Coronary artery disease	406,323 (42.5)	152,115 (38.7)	7.8	558,438 (41.4)
Previous myocardial infarction	146,515 (15.3)	50,590 (12.9)	7.1	197,105 (14.6)
Previous PCI	72,223 (7.6)	28,619 (7.3)	1.1	100,842 (7.5)
Previous CABG	60,510 (6.3)	21,370 (5.4)	3.8	81,880 (6.1)
Mitral regurgitation	101,891 (10.7)	40,145 (10.2)	1.5	142,036 (10.5)
Aortic regurgitation	38,757 (4.1)	16,438 (4.2)	−0.6	55,195 (4.1)
Aortic stenosis	100,671 (10.5)	41,234 (10.5)	0.2	141,905 (10.5)
Vascular disease	256,016 (26.8)	107,795 (27.4)	−1.4	363,811 (27.0)
Pacemaker or ICD	97,026 (10.1)	55,890 (14.2)	−12.4	152,916 (11.3)
ICD	12,705 (1.3)	1259 (0.3)	11.2	13,964 (1.0)
CRT	6864 (0.7)	661 (0.2)	8.3	7525 (0.6)
Ischemic stroke	54,539 (5.7)	37,338 (9.5)	−14.3	91,877 (6.8)
Alcohol related diagnoses	57,453 (6.0)	24,196 (6.1)	−0.6	81,649 (6.0)
Abnormal renal function	111,350 (11.6)	52,274 (13.3)	−5.0	163,624 (12.1)
Lung disease	222,083 (23.2)	101,327 (25.7)	−5.9	323,410 (24.0)
Sleep apnea syndrome	64,487 (6.7)	32,811 (8.3)	−6.0	97,298 (7.2)
COPD	132,935 (13.9)	59,555 (15.1)	−3.5	192,490 (14.3)
Liver disease	47,817 (5.0)	21,837 (5.5)	−2.4	69,654 (5.2)
Thyroid diseases	105,783 (11.1)	64,707 (16.4)	−15.7	170,490 (12.6)
Inflammatory disease	71,214 (7.4)	39,076 (9.9)	−8.8	110,290 (8.2)
Anemia	184,967 (19.3)	103,148 (26.2)	−16.4	288,115 (21.3)
Previous cancer	151,136 (15.8)	84,819 (21.6)	−14.8	235,955 (17.5)
Poor nutrition	109,402 (11.4)	65,127 (16.5)	−14.8	174,529 (12.9)
Cognitive impairment	117,339 (12.3)	69,280 (17.6)	−15.0	186,619 (13.8)

AF, atrial fibrillation; CABG, coronary artery bypass graft; COPD, chronic obstructive pulmonary disease; CRT, cardiac re-synchronization therapy; HF, heart failure; ICD, implantable cardioverter defibrillator; PCI, percutaneous coronary intervention.

**Table 2:** Rate of medication at discharge for patients with AF and HF (at the time of hospitalization with the last event between AF or HF).

	Prevalent HF	Incident HF
	(*n* = 9352)	(*n* = 4248)
ACE inhibitor or ARB	4142 (44.3%)	1635 (38.5%)
Beta-blocker	4577 (48.9%)	1998 (47.0%)
Diuretic	5384 (57.6%)	2371 (55.8%)
K-sparing diuretics	1039 (11.1%)	375 (8.8%)
Calcium channel blocker	1497 (16.0%)	764 (18.0%)
Digoxin	795 (8.5%)	378 (8.9%)
Anti-arrhythmic agents	2631 (28.1%)	994 (23.4%)
Amiodarone	2494 (26.7%)	907 (21.4%)
VKA	3362 (35.9%)	1596 (37.6%)
Direct oral anti-coagulant	1532 (16.4%)	740 (17.4%)
Dabigatran	242 (2.6%)	124 (2.9%)
Rivaroxaban	617 (6.6%)	321 (7.6%)
Apixaban	691 (7.4%)	310 (7.3%)
Aspirin	2462 (26.3%)	919 (21.6%)
P2Y12 inhibitor	801 (8.6%)	279 (6.6%)
Statin	2898 (31.0%)	1121 (26.4%)
Antidiabetic	1677 (17.9%)	757 (17.8%)

% of use for each medication was identified in the Echantillon Généraliste des Bénéficiaires (EGB) permanent random sample (1/97) of the French nationwide claims database for patients with same inclusion criteria (*n* = 13,600 patients with AF and HF). ACE, angiotensin-converting enzyme; AF, atrial fibrillation; ARB, angiotensin receptor blocker; HF, heart failure.

**Table 3:** Baseline characteristics of matched patients with AF and prevalent HF or incident HF.

	Prevalent HF	Incident HF	Standardized Difference, Prevalent HF vs. Incident HF
	(*n* = 212,293)	(*n* = 212,293)	(*n* = 212,293)
Age, years	81.5 ± 9.8	81.4 ± 10.0	−1.1
Sex (male)	101,838 (48.0)	102,423 (48.2)	0.9
CHA_2_DS_2_-VASc score	4.5 ± 1.2	4.5 ± 1.3	0.3
HASBLED score	1.9 ± 0.7	1.9 ± 0.7	0.6
Hypertension	159,619 (75.2)	160,555 (75.6)	1.0
Diabetes mellitus	45,788 (21.6)	47,155 (22.2)	1.5
Smoker	8091 (3.8)	8926 (4.2)	1.5
Dyslipidemia	48,405 (22.8)	50,225 (23.7)	1.9
Obesity	30,053 (14.2)	30,939 (14.6)	1.1
History of pulmonary edema	5751 (2.7)	6750 (3.2)	2.0
Dilated cardiomyopathy	21,158 (10.0)	22,755 (10.7)	2.1
Coronary artery disease	70,625 (33.3)	72,363 (34.1)	1.7
Previous myocardial infarction	22,837 (10.8)	24,018 (11.3)	1.6
Previous PCI	11,389 (5.4)	12,098 (5.7)	1.3
Previous CABG	7865 (3.7)	8183 (3.9)	0.6
Mitral regurgitation	14,108 (6.6)	14,734 (6.9)	1.0
Aortic regurgitation	4493 (2.1)	4811 (2.3)	0.8
Aortic stenosis	14,791 (7.0)	15,659 (7.4)	1.3
Vascular disease	41,500 (19.5)	43,398 (20.4)	2.0
Pacemaker or ICD	18,855 (8.9)	19,279 (9.1)	0.6
ICD	282 (0.1)	308 (0.1)	0.1
CRT	129 (0.1)	142 (0.1)	0.1
Ischemic stroke	11,027 (5.2)	11,630 (5.5)	1.1
Alcohol related diagnoses	6062 (2.9)	6738 (3.2)	1.3
Abnormal renal function	17,156 (8.1)	18,138 (8.5)	1.4
Lung disease	39,514 (18.6)	40,366 (19.0)	0.9
Sleep apnea syndrome	9262 (4.4)	9878 (4.7)	1.1
COPD	23,494 (11.1)	23,993 (11.3)	0.7
Liver disease	5139 (2.4)	5682 (2.7)	1.1
Thyroid diseases	23,649 (11.1)	23,923 (11.3)	0.4
Inflammatory disease	11,570 (5.5)	11,901 (5.6)	0.6
Anemia	38,038 (17.9)	39,034 (18.4)	1.1
Previous cancer	33,252 (15.7)	34,512 (16.3)	1.5
Poor nutrition	23,329 (11.0)	24,008 (11.3)	0.9
Cognitive impairment	29,406 (13.9)	28,925 (13.6)	−0.6

AF, atrial fibrillation; CABG, coronary artery bypass graft; COPD, chronic obstructive pulmonary disease; CRT, cardiac re-synchronization therapy; HF, heart failure; ICD, implantable cardioverter defibrillator; PCI, percutaneous coronary intervention.

**Table 4:** Clinical outcomes during the whole follow-up in the unmatched cohort of patients with AF and prevalent or incident HF with follow-up starting at the time of last event between AF or HF.

	Prevalent HF (*n* = 956,086)	Incident HF (*n* = 393,552)	HR (95% CI) for Prevalent HF vs. Incident HF	*p* (Uncorrected)	*p* (Bonferroni Correction)
All-cause death	316,729 (19.0)	126,688 (21.8)	0.91 (0.91–0.92)	<0.0001	<0.0001
Cardiovascular death	117,294 (7.1)	41,431 (7.1)	1.04 (1.03–1.06) *	<0.0001	<0.0001
Non-cardiovascular death	199,435 (12.0)	85,257 (14.7)	0.85 (0.84–0.86)	<0.0001	<0.0001
Ischemic stroke	40,670 (2.5)	14,266 (2.6)	0.97 (0.95–0.99) ^†^	0.006	0.02
Re-hospitalization for HF	254,885 (19.3)	45,877 (13.8)	1.43 (1.42–1.45)	<0.0001	<0.0001
Cancer	73,528 (4.7)	25,214 (4.9)	0.96 (0.94–0.97)	<0.0001	<0.0001

* hazard ratio = 1.10 (1.08–1.11), uncorrected *p* < 0.0001, corrected *p* < 0.0001 by Fine and Gray model for competing risks of cardiovascular and non-cardiovascular death. ^†^ hazard ratio = 1.04 (1.02–1.06), uncorrected *p* < 0.0001, corrected *p* < 0.0001 by Fine and Gray model for competing risks of ischemic stroke and death. Values are n (incidence rate, %/year). AF, atrial fibrillation; CI, confidence interval; HF, heart failure; HR, hazard ratio.

**Table 5:** Clinical outcomes during the whole follow-up in the matched cohort of patients with AF and prevalent or incident HF with follow-up starting at the time of last event between AF or HF.

	Prevalent HF (*n* = 212,293)	Incident HF (*n* = 212,293)	HR (95% CI) for Prevalent HF vs. Incident HF	*p* (Uncorrected)	*p* (Bonferroni Correction)
All-cause death	68,150 (21.6)	64,643 (19.3)	1.10 (1.08–1.11)	<0.0001	<0.0001
Cardiovascular death	24,156 (7.7)	21,874 (6.5)	1.14 (1.12–1.16) *	<0.0001	<0.0001
Non-cardiovascular death	43,994 (14.0)	42,769 (12.8)	1.07 (1.06–1.09)	<0.0001	<0.0001
Ischemic stroke	3775 (1.2)	7824 (2.4)	0.50 (0.48–0.52) ^†^	<0.0001	<0.0001
Re-hospitalization for HF	49,763 (19.4)	24,785 (13.2)	1.44 (1.41–1.46)	<0.0001	<0.0001
Cancer	14,558 (4.9)	15,176 (5.0)	0.97 (0.95–0.99)	0.005	0.02

* hazard ratio = 1.13 (1.11–1.16), uncorrected *p* < 0.0001, corrected *p* < 0.0001 by Fine and Gray model for competing risks of cardiovascular and non-cardiovascular death. ^†^ hazard ratio = 0.48 (0.46–0.50), uncorrected *p* < 0.0001, corrected *p* < 0.0001 by Fine and Gray model for competing risks of ischemic stroke and death. Values are n (incidence rate, %/year). AF, atrial fibrillation; CI, confidence interval; HF, heart failure; HR, hazard ratio.

## Data Availability

The data and study materials will not be made available to other researchers for purposes of reproducing the results or replicating the procedure. Because this study used data from human subjects, the data and everything pertaining to the data are governed by the French Health Agencies and cannot be made available to other researchers.
